# Nf1 RasGAP Inhibition of LIMK2 Mediates a New Cross-Talk between Ras and Rho Pathways

**DOI:** 10.1371/journal.pone.0047283

**Published:** 2012-10-17

**Authors:** Béatrice Vallée, Michel Doudeau, Fabienne Godin, Aurélie Gombault, Aurélie Tchalikian, Marie-Ludivine de Tauzia, Hélène Bénédetti

**Affiliations:** Centre de Biophysique Moléculaire, Centre Nationale de la Recherche Scientifique (CNRS), University of Orléans and Institut National de la Santé et de la Recherche Médicale (INSERM), Orléans, France; Institute of Developmental Biology and Cancer Research, France

## Abstract

**Background:**

Ras GTPases mediate numerous biological processes through their ability to cycle between an inactive GDP-bound form and an active GTP-bound form. Guanine nucleotide exchange factors (GEFs) favor the formation of the active Ras-GTP, whereas GTPase activating proteins (GAPs) promote the formation of inactive Ras-GDP. Numerous studies have established complex signaling cross-talks between Ras GTPases and other members of the superfamily of small GTPases. GEFs were thought to play a major role in these cross-talks. However, recently GAPs were also shown to play crucial roles in these processes. Among RasGAPs, Nf1 is of special interest. Nf1 is responsible for the genetic disease Neurofibromatosis type I, and recent data strongly suggest that this RasGAP connects different signaling pathways.

**Methodology/Principal Findings:**

In order to know if the RasGAP Nf1 might play a role in connecting Ras GTPases to other small GTPase pathways, we systematically looked for new partners of Nf1, by performing a yeast two-hybrid screening on its SecPH domain. LIMK2, a major kinase of the Rho/ROCK/LIMK2/cofilin pathway, was identified in this screening. We confirmed this interaction by co-immunoprecipitation experiments, and further characterized it. We also demonstrated its specificity: the close related homolog of LIMK2, LIMK1, does not interact with the SecPH domain of Nf1. We then showed that SecPH partially inhibits the kinase activity of LIMK2 on cofilin. Our results furthermore suggest a precise mechanism for this inhibition: in fact, SecPH would specifically prevent LIMK2 activation by ROCK, its upstream regulator.

**Conclusions/Significance:**

Although previous data had already connected Nf1 to actin cytoskeleton dynamics, our study provides for the first time possible detailed molecular requirements of this involvement. Nf1/LIMK2 interaction and inhibition allows to directly connect neurofibromatosis type I to actin cytoskeleton remodeling, and provides evidence that the RasGAP Nf1 mediates a new cross-talk between Ras and Rho signaling pathways within the superfamily of small GTPases.

## Introduction

Ras GTPases act as molecular switches cycling between an inactive GDP bound form and an active GTP bound form. In response to various extracellular stimuli, the activated form of Ras GTPases interacts with specific downstream effectors thus regulating many major cellular processes, such as cell proliferation and differentiation, morphology, migration, and apoptosis. GDP/GTP cycling is controlled by two categories of proteins. Guanine nucleotide exchange factors (GEFs) catalyze the release of GDP thus allowing the binding of GTP, whereas GTPase Ativating Proteins (GAPs) enhance intrinsic Ras GTPase activity thus promoting hydrolysis of GTP into GDP.

RasGEFs have been extensively studied, and their connections with different signaling pathways have been well established [1]. In contrast, RasGAPs have received relatively little attention and there is less information regarding their regulation. However, emerging pieces of evidence show that RasGAP interaction with other partners mediates cross-talk between Ras GTPases and other small GTPase signaling pathways. Along this line, p120 RasGAP was shown to interact with and to influence the activity of several RhoGAPs: p190 RhoGAP, p200 RhoGAP, and DLC1 RhoGAP [2], [3]. Beside p120 RasGAP, various other mammalian RasGAPs have been identified, including neurofibromin, RASA2, IQGAP1, IQGAP3, SYNGAP and GAPVD1 [4]. However, only mutations in p120 RasGAP and neurofibromin result in a clinical expression and lead to human hereditary disorders.

Neurofibromin (Nf1) is encoded by *NF1* gene which has been identified as a tumor suppressor gene involved in Neurofibromatosis type I. Neurofibromatosis type I (NF1), also known as von Recklinghausen disease, is an autosomal dominant disorder and one of the most common genetic diseases as it affects 1 individual in 3,500. The phenotype of NF1 is highly variable: “café au lait” spots on the skin, iris Lish nodules, and bone deformations are often encountered. However, the hallmark of NF1 is the development of nerve tumors with an increased risk of malignancies, and neurological disorders such as learning disabilities [5], [6], [7]. NF1 is due to mutations within the *NF1* gene which encodes neurofibromin, a large 2818 amino acid protein [8], [9], [10]. Initially, sequence analysis of neurofibromin revealed a GAP Related Domain (GRD) with high identity (31%) with the GAP domain of p120RasGAP. Biochemical studies confirmed that Nf1 has Ras-GAP activity [11], [12], [13]. Therefore, primary studies have focused on Ras regulation by Nf1. Loss or mutations of Nf1 in a wide variety of both human tumors and *NF1*-deficient mice result in increased levels of active Ras-GTP and consequently activate various Ras effectors thereby promoting cell proliferation and differentiation [14], [15]. However, recent data show that, besides regulating Ras, Nf1 plays a critical role in other signaling pathways. Indeed, Nf1 has been shown to be involved in the regulation of intracellular cAMP in human [16], mouse [17], [18], [19], drosophila [20], [21], and yeast [22]. On the other hand, Nf1 was also suggested to play a role in actin cytoskeleton remodeling. Indeed, Nf1 was demonstrated to regulate cell adhesion, cell motility and actin cytoskeleton reorganisation [23], [24], [25]. Nf1 was shown to associate with microtubular and microfilamentous cytoskeleton [26], and to interact with FAK (Focal Adhesion Kinases) [27] and syndecans thus modulating PKA-Ena/VASP pathway in the formation of filopodia and dendritic spine [28]. Nf1 was also shown to enhance cell motility by regulating actin filament dynamics *via* the inhibition of the Rho/ROCK/LIMK2/cofilin pathway [29]. Furthermore, Nf1 was shown to act as a negative regulator of the Rac1/Pak1/LIMK1/cofilin pathway independently of Ras signaling pathways [30]. Although Nf1 involvement in these different signaling pathways is now well established, most of its molecular targets are still unknown, and the molecular mechanisms of these involvements remain in most cases to be elucidated.

As the RasGAP Nf1 seems to connect several signal transduction pathways, it appears as a good candidate to link Ras GTPases to other small GTPase pathways. In this context, we decided to systematically look for new partners of Nf1 by performing a two-hybrid screening. We focused on a specific domain of Nf1, SecPH. We chose this domain as it appeared well appropriate for our study. Firstly, Sec and PH domains are well known to mediate protein-protein interactions. Secondly, Nf1 SecPH 3D structure has been resolved revealing a well defined structure *per se* [31].Thirdly, SecPH flanks the GRD of Nf1 and could regulate its activity in an allosteric way. Finally, we already demonstrated in yeast that this domain is able to mediate protein-protein interactions [32].

Our two-hybrid screening allowed us to identify LIMK2 as a partner of Nf1-SecPH. LIMK2 is a major kinase in the Rho/ROCK/LIMK2/cofilin pathway. This pathway plays a major role in actin cytoskeleton remodeling. We confirmed the interaction between Nf1-SecPH and LIMK2 by coimmunoprecipitation experiments and dissected the molecular requirements of this interaction. From a functional point of view, our data showed that SecPH is an inhibitor of LIMK2: in the presence of SecPH, LIMK2 phosphorylates less efficiently its substrate cofilin. Our experiments also strongly suggested that SecPH interferes with LIMK2 activation by ROCK (Rho-associated coiled-coil-forming protein kinase): SecPH specifically prevents LIMK2 phosphorylation by this upstream kinase. Our findings therefore propose a molecular explanation for the connection between Nf1 and actin cytoskeleton remodelling, and thus shed light on a new cross-talk between Ras and Rho signaling pathways.

## Results

### Two-hybrid screening results

**Figure 1:**
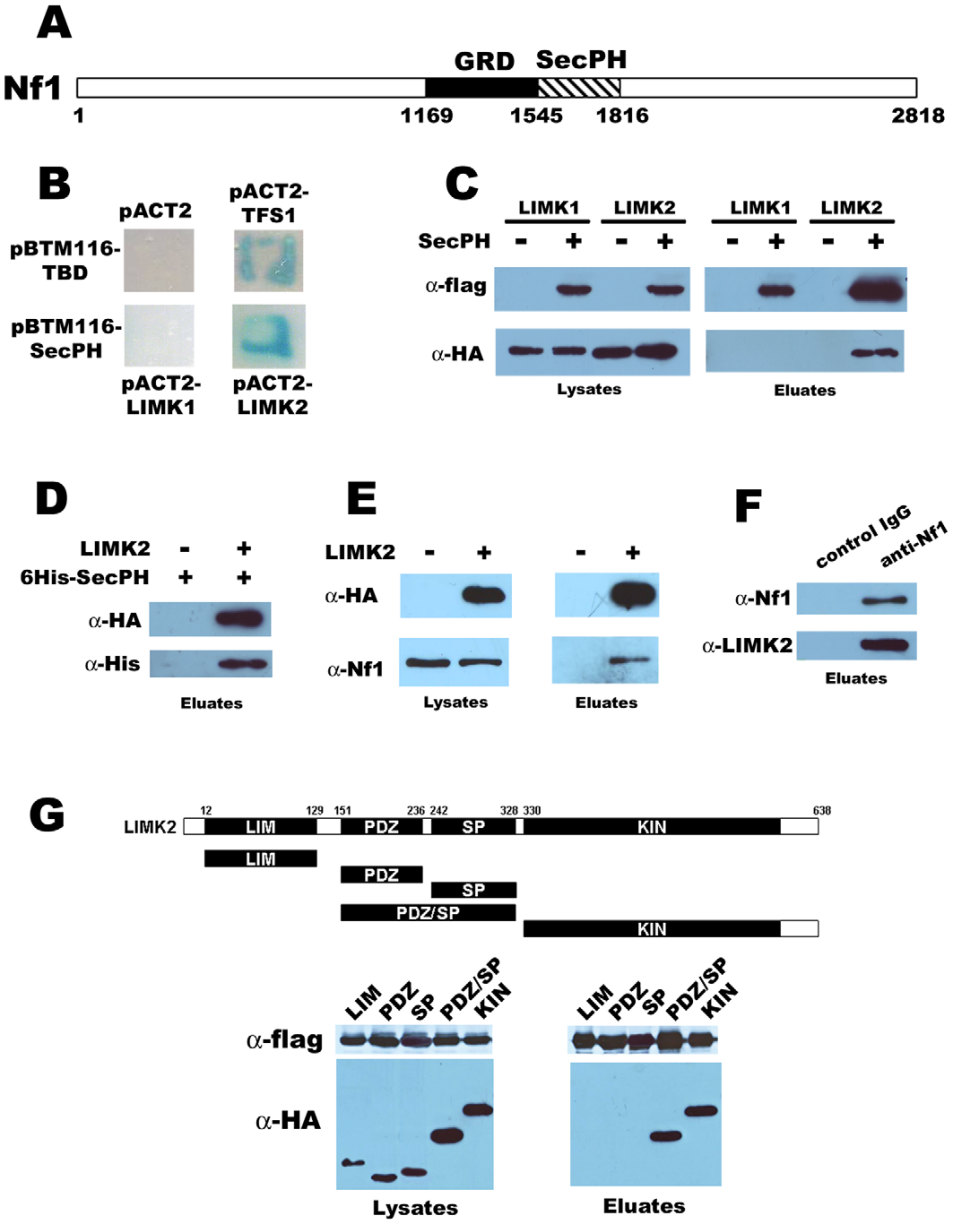
Interaction between LIMK2 and the SecPH domain of Nf1. *A. Diagram of Nf1.* GRD (GAP related domain) responsible for the main known function of Nf1 is depicted as well as SecPH, the region used for the two-hybrid screening. *B. Interaction revealed by the two-hybrid screening.* L40 cells transformed with pBTM116-TBD were mated with Y187 cells transformed with the empty plasmid pACT2 (as a negative control) or pACT2-TFS1 (as a positive control, as demonstrated by [32]). L40 cells transformed with pBTM116-SecPH were mated with Y187 cells transformed with pACT2-LIMK1 or pACT2-LIMK2. After mating on YPD, the resultant diploids were selected on a SD-LW medium. The interaction between the LexA fusion proteins encoded by the pACT2 plasmids and the Gal4 fusion proteins encoded by pBTM116 plasmids was tested by checking the growth of diploids on a SD-LWH media containing 3AT (1 mM) and their ability to cleave X-gal (1 mM) thereby attesting the production of β-galactosidase. *C. Interaction in HEK-293 transfected cells.* HEK-293 cells were cotransfected with either HA-LIMK2 or HA-LIMK1 and flag-SecPH or its parental empty plasmid (p3XFlag). Cell lysates and anti-flag immunoprecipitation eluates were analyzed by immunobloting. *D. Immunoprecipitated LIMK2 interacts with recombinant 6His-SecPH.* HEK-293 cells were transfected with HA-LIMK2 or its parental empty plasmid, pcDNA3. The corresponding cell lysates were immunoprecipitated with anti-HA beads. Beads were then incubated with 6His-SecPH in lysis buffer. Anti-HA immunoprecipitates were analyzed by immunobloting. *E. Transfected LIMK2 interacts with endogenous Nf1.* Cells were transfected with HA-LIMK2 or its parental empty plasmid, pcDNA3. Lysates and anti-HA immunoprecipitates were analyzed by immunobloting. *F. Endogenous LIMK2 interacts with endogenous Nf1* Anti-Nf1 immunoprecipitates from HEK-293 were analyzed by immunobloting. *G. Domains of LIMK2 involved in its interaction with SecPH*. Top. Schematic diagram of LIMK2 and its various fragments designed for this study. Bottom. Cells were cotransfected with SecPH and one of the domains of LIMK2. Lysates and anti-flag immunoprecipitates were analyzed by immunobloting.

In an effort to identify a connection between Ras GTPases and other small GTPases *via* the RasGAP Nf1, we decided to systematically look for new partners of the RasGAP Nf1. In this purpose, we performed a yeast two-hybrid screening of a human fetal brain cDNA library on a newly characterized domain of Nf1, SecPH [31] (Figure 1A). This screening was particularly successful as we could test 270 million of interactions, which means a recovery of the library of 70 times for a final number of 1464 positive candidates. Among these 1464 candidates, we identified LIMK2 (Figure 1B).

LIMK2 is a serine threonine kinase playing a major role in actin cytoskeleton dynamics *via* the Rho/ROCK/LIMK2/cofilin signaling pathway. LIMK2, and its sole homolog LIMK1, have a unique organization of signaling domains, with two amino-terminal LIM domains (each containing double zinc finger motifs), adjacent PDZ and serine/proline (SP)-rich regions, followed by a carboxy-terminal kinase domain. Upon their activation by ROCK, LIMK2 and LIMK1 phosphorylate cofilin, resulting in its inactivation. Cofilin is a member of ADF (actin depolymerizing factor) family. It promotes actin depolymerization at pointed ends and severes long actin filaments, which leads to a fast turnover of actin filaments [33], [34]. By inhibiting cofilin, LIMK2 and LIMK1 play a central role in the regulation of actin cytoskeleton.

### LIMK2 interacts with the SecPH domain of Nf1

To validate the interaction found by our two-hybrid screening between SecPH and LIMK2 (Figure 1B), we performed coimmunoprecipitation experiments. On the one hand LIMK2 was tagged by a HA epitope, on the other hand SecPH was tagged with a flag epitope. HEK-293 cells were cotransfected by LIMK2 and SecPH or its parental empty plasmid. Anti-flag immunoprecipitations were performed. Lysates and eluates were subjected to western blot analysis. As shown in Figure 1C, LIMK2 specifically coimmunoprecipitated with SecPH. Indeed, no LIMK2 was detected in the anti-flag-immunoprecipitates using cells transfected with the parental empty plasmid of SecPH (p3XFlag). Therefore, in transfected HEK293 cells, LIMK2 specifically interacts with SecPH.

To further study the interaction between LIMK2 and SecPH, recombinant 6His-SecPH was incubated with immunoprecipitated HA-LIMK2 for a complex formation assay. As shown in Figure 1D, recombinant 6His-SecPH is co-purified with immunoprecipitated HA-LIMK2.

### SecPH does not interact with LIMK2 close related homolog, LIMK1

LIMK2 has a sole homolog, LIMK1. They share 50% identity. In our two-hybrid screening, we could identify LIMK2 but not LIMK1. As LIMK1 and LIMK2 are very close we wondered if the SecPH-LIMK2 interaction could be conserved with LIMK1.

Using the two-hybrid system, we could detect no interaction between LIMK1 and SecPH (Figure 1B).

By coimmunoprecipitation experiments on lysates of cells cotransfected with SecPH and LIMK1, no interaction could either be detected (Figure 1C).

In conclusion, SecPH binds to LIMK2 but not to its related homolog LIMK1.

### LIMK2 interacts with endogenous Nf1

We have shown that LIMK2 is able to interact with SecPH, a domain of Nf1. We wondered if this interaction was still observed with the full length protein Nf1.

To address this point, we first transfected HEK-293 cells, which naturally express Nf1, with HA-LIMK2, and proceeded to anti-HA immunoprecipitation. As shown in Figure 1E, immunoprecipitated HA-LIMK2 interacts specifically with endogenous Nf1.

We then immunoprecipitated endogenous Nf1 from HEK-293 lysed cells with anti-Nf1 antibodies coupled to sepharose beads and checked for endogenous LIMK2 interaction. As shown in Figure 1F, we could detect a specific band of endogenous LIMK2 interacting with endogenous Nf1.

### Domains of LIMK2 involved in its interaction with SecPH

To further dissect the regions of LIMK2 involved in its interaction with SecPH, different domains of LIMK2 were tested for their abilities to coimmunoprecipitate with SecPH. Five domains of LIMK2 were tested (Figure 1G - Top): LIM (the N-terminal extremity of LIMK2 containing 2 LIM domains), PDZ, SP, PDZ/SP, and KIN (the C-terminal domain of LIMK2 comprising the kinase domain).

As shown in Figure 1G - Bottom, LIM domain is unable to interact with SecPH, neither PDZ nor SP domains. In contrast, KIN and PDZ/SP domains are able to interact with SecPH. It is quite intriguing that SecPH interacts with the double domain PDZ/SP but not with one of these single domains PDZ or SP, this may be explained by conformational requirements.

Therefore, SecPH interacts with LIMK2 *via* two domains: the kinase and the PDZ/SP domains.

### SecPH affects LIMK2 induced formation of actin stress fibers

LIMK2 belongs to the Rho/ROCK/LIMK2/cofilin signal transduction pathway and phosphorylates cofilin. Once phosphorylated, cofilin, an actin depolymerisation factor, is then no longer able to depolymerise actin, and an accumulation of stress fibers is observed. Using *NF1* siRNA, Ozawa *et al.* have shown that Nf1 is an inhibitor of this pathway [29]. However, they provided no molecular explanation for this phenomenon. Our new data have prompted us to test if the target of Nf1 in the Rho/ROCK/LIMK2/cofilin pathway could be LIMK2.

We first focused our attention on actin cytoskeleton organisation driven by this pathway by performing immunofluorescence experiments on fixed intact cells.

**Figure 2:**
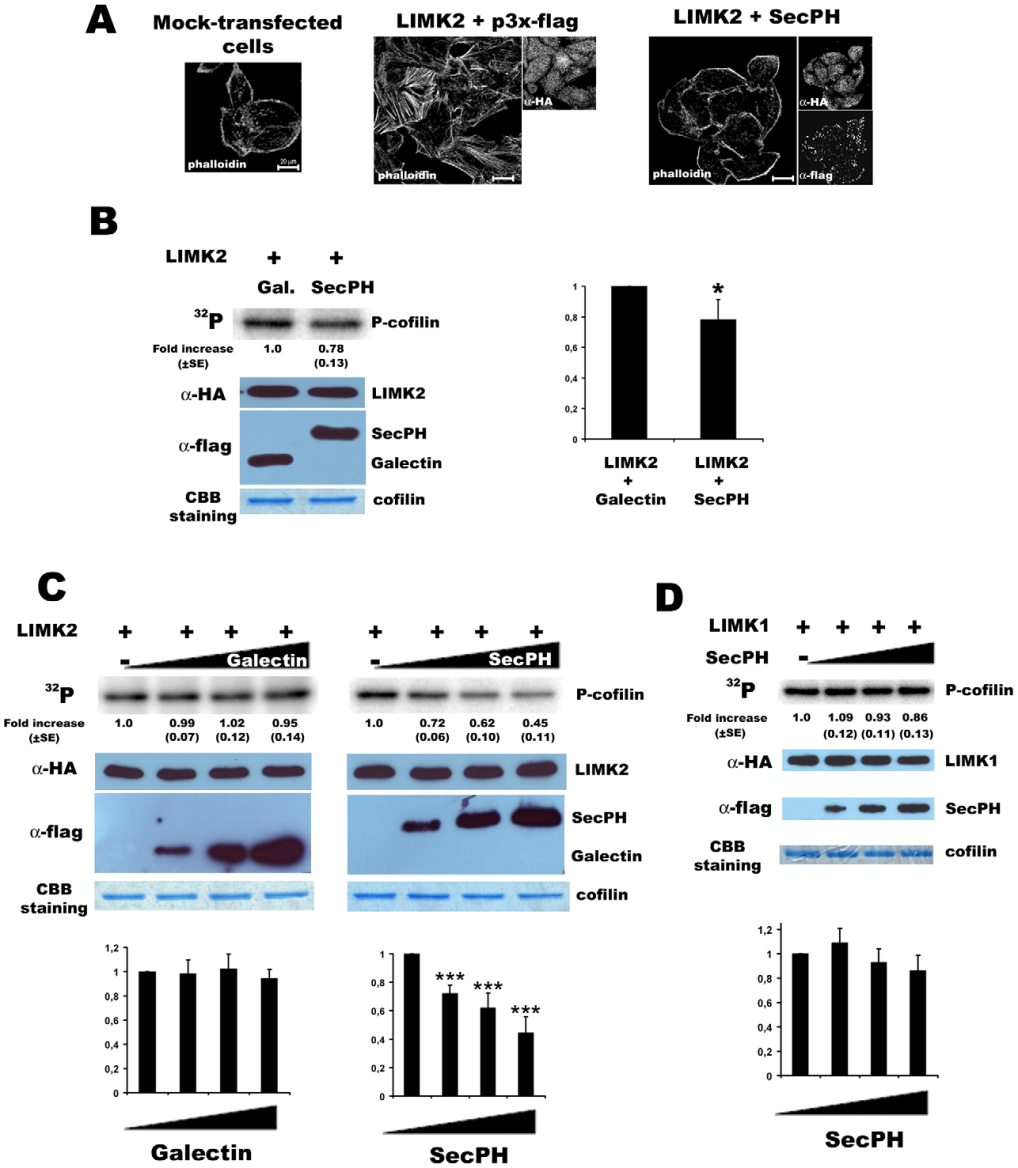
SecPH partially inhibits cofilin phosphorylation by LIMK2, but not by LIMK1. *A*. *Actin cytoskeleton organisation*. HeLa cells were cotransfected with pcDNA3, LIMK2 and SecPH or its parental empty plasmid, p3XFlag. Cells were fixed and stained with phalloidin, or anti-HA or anti-flag antibodies. *B. Inhibition of LIMK2 cofilin phosphorylation by SecPH.* Cells were cotransfected with LIMK2 and SecPH or Galectin-3 (a non-specific control protein). Immunoprecipitated HA-LIMK2 and GST-cofilin were used in the kinase assay. The kinase activity on cofilin of immunoprecipitated HA-LIMK2 from cells cotransfected with Galectin-3 was taken as 1.0. Each value represents the mean ± SE (standard error) of four independent experiments. Statistical significance was determined relative to control using one-way ANOVA (* p<0.05). The HA-immunoprecipitates were also submitted to HA-immunoblotting and to coomassie blue staining. Lysates were submitted to flag-immunoblotting. *C. Dose dependent inhibition of LIMK2 cofilin phosphorylation by SecPH.* Cells were transfected with LIMK2 and either SecPH or Galectin-3. SecPH and Galectin-3 cell lysates were immunoprecipitated with anti-flag beads, beads were then eluted flag peptide. Immunoprecipitated HA-LIMK2 and GST-cofilin were used for kinase assay and were incubated with increasing amount of immunoprecipitated SecPH or Galectin-3 (0, 6, 12, 18 ul respectively). The kinase activity on cofilin of immunoprecipitated HA-LIMK2 with no addition of immunoprecipitated SecPH or Galectin-3 was taken as 1.0. Each value represents the mean ± SE of four independent experiments. Statistical significance was determined relative to control using one-way ANOVA (*** p<0.0001). Immunoprecipitates were also subjected to immunoblotting and to coomassie blue staining. *D. SecPH does not inhibit cofilin phosphorylation by LIMK1.* Cells were transfected either with SecPH or with LIMK1. SecPH cell lysates were immunoprecipitated with anti-flag beads, beads were then eluted with flag peptide. Immunoprecipitated HA-LIMK1 was used for kinase assay and was incubated with increasing amount of immunoprecipitated SecPH (0, 6, 12, 18 ul respectively). The kinase activity on cofilin of immunoprecipitated HA-LIMK1 with no addition of immunoprecipitated SecPH was taken as 1.0. Each value represents the mean ± SE of two independent experiments. Immunoprecipitates were also subjected to immunoblotting and to coomassie blue staining.

HeLa cells were cotransfected with HA-LIMK2 and SecPH or with HA-LIMK2 and the parental empty plasmid of SecPH. The actin filaments were visualized by phalloidin staining. As previously observed [35], expression of LIMK2 enhanced the formation of actin stress fibers compared to mock-transfected cells, a consequence of cofilin inactivation by LIMK2 (Figure 2A- Left and Middle pannels). When SecPH was cotransfected with LIMK2, there was a marked decrease in the accumulation of actin stress fibers (Figure 2A – Right and Middle pannels). These data suggest that SecPH affects actin stress fiber formation due to LIMK2 overexpression.

### SecPH partially inhibits cofilin phosphorylation by LIMK2

We then decided to test directly SecPH activity on LIMK2 by measuring LIMK2 kinase activity on cofilin *in vitro* in the presence or not of SecPH.

Cells were cotransfected by LIMK2 and either SecPH or Galectin-3, a non-specific flag-tagged protein control. Galectin-3 is a member of the lectin family which binds beta-galactoside [36]. We measured the kinase activity of the anti-HA-LIMK2 immunoprecipitates using recombinant GST-cofilin as a substrate.

As shown in Figure 2B, anti-HA immunoprecipitate from cells cotransfected with LIMK2 and SecPH showed a slight but significant and reproducible decrease of around 20% in the intensity of phospho-cofilin (P-cofilin) compared with anti-HA immunoprecipitate from cells cotransfected with LIMK2 and the non-specific protein control Galectin-3. These data are statistically significant as p = 0.0289 (* p<0.05).

These results suggest that SecPH partially inhibits cofilin phosphorylation by LIMK2.

### SecPH dose dependent inhibition of cofilin phosphorylation by LIMK2

To further characterize SecPH inhibition of cofilin phosphorylation by LIMK2, we repeated the kinase assay on cofilin, but this time we incubated anti-HA-LIMK2 immunoprecipitate with increasing amounts of immunoprecipitated SecPH. Cells were transfected on one hand with LIMK2 and on the other hand with SecPH or the non-specific protein control Galectin-3. SecPH and Galectin-3 were immunoprecipitated from transfected cell lysates with anti-flag beads, and then eluted from the beads with flag peptide. For the kinase assay, immunoprecipitated HA-LIMK2 and GST-cofilin were used, in the presence of increasing amounts of eluted SecPH or Galectin-3.

As shown in Figure 2C, we observed a dose-dependent response of cofilin phosphorylation upon SecPH addition, whereas Galectin-3 addition had no influence on cofilin phosphorylation. The more SecPH was added, the less P-cofilin was observed. Therefore, the more SecPH was added, the more LIMK2 kinase activity on cofilin was inhibited.

### SecPH inhibition of cofilin phosphorylation is specific to LIMK2

We already demonstrated that SecPH specifically interacts with LIMK2 and not with its close related homolog LIMK1 (Figures 1B and 1C). In order to make sure that SecPH activity on cofilin phosphorylation *in vitro* specifically went through LIMK2, and not through its close related homolog, LIMK1, we repeated the kinase assay on GST-cofilin with HA-LIMK1 immunoprecipitate in the presence of increasing amounts of immunoprecipitated SecPH. It appeared that SecPH has no effect on LIMK1 kinase activity on cofilin (Figure 2D).

### SecPH inhibition of cofilin phosphorylation by LIMK2 requires ROCK activation of LIMK2

To deepen our understanding of SecPH inhibition on cofilin phosphorylation by LIMK2, we took advantage of two mutants of LIMK2 at Threonin 505. Consequently to T505 phosphorylation by ROCK, LIMK2 is activated and phosphorylates cofilin. The two mutants used in this study were LIMK2-T505A, which is inactive, and LIMK2-T505EE, which is constitutively active [35].

**Figure 3:**
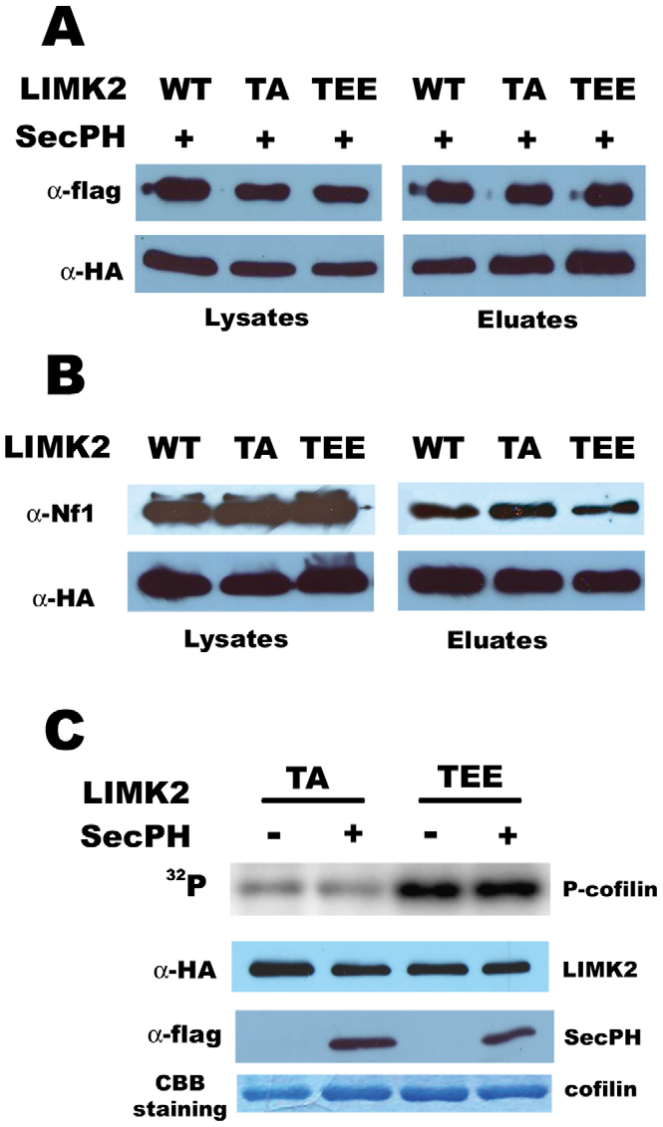
SecPH inhibition of cofilin phosphorylation by LIMK2 requires ROCK activation of LIMK2. *A. SecPH interacts with LIMK2 whatever its activation state.* Cells were cotransfected with SecPH and either LIMK2-WT, or LIMK2-TA or LIMK2-TEE. Lysates and anti-flag immunoprecipitates were subjected to immunoblotting. *B. Nf1 interacts with LIMK2 whatever its activation state.* Cells were transfected with either LIMK2-WT, or LIMK2-TA or LIMK2-TEE. Lysates and anti-HA immunoprecipitates were subjected to immunoblotting. *C. SecPH is unable to modulate cofilin phosphorylation by LIMK2-T505 mutants*. Cells were cotransfected with either LIMK2-WT, or LIMK2-TA or LIMK2-TEE and SecPH or its parental empty plasmid. Immunoprecipitated HA-LIMK2 and GST-cofilin were used in the kinase assay. Anti-HA immunoprecipitates were also subjected to immunoblotting and to coomassie blue staining.

First, we checked if these two mutants were still able to interact with SecPH. Cells were cotransfected with HA-LIMK2 WT, LIMK2-TA or LIMK2-TEE and with flag-SecPH. Anti-flag immunoprecipitations were performed. As depicted in Figure 3A, SecPH interacts with wild-type LIMK2, as well as with LIMK2-TA and LIMK2-TEE. We also checked if these two mutants were still able to interact with endogenous Nf1. Cells were transfected with either HA-LIMK2-WT, or LIMK2-TA or LIMK2-TEE. Anti-HA immunoprecipitations were performed. As shown in Figure 3B, the two mutants interact with endogenous Nf1.

We then tested the ability of SecPH to inhibit cofilin phosphorylation by these two mutants. Cells were cotransfected by SecPH or its empty parental plasmid and by either LIMK2-WT, LIMK2-TA, or LIMK2-TEE. We measured the kinase activity of the different anti-HA-LIMK2 immunoprecipitates using GST-cofilin as a substrate. P-cofilin signal was very weak with the inactive LIMK2-TA and particularly intense with the constitutively active LIMK2-TEE (Figure 3C). These results are in good accordance with data from the literature [35]. Neither of these P-cofilin signals was modulated by SecPH (Figure 3C). These results are not so surprising for P-cofilin produced by LIMK2-TA, considering the mutant has a weak activity. SecPH was interestingly unable to inhibit cofilin phosphorylation by the constitutively active LIMK2-TEE whereas it can still interact with it (Figure 3A).

As the constitutively active mutant LIMK2-TEE bypasses ROCK activation of LIMK2, these results suggest that SecPH might act upstream from LIMK2 and that its inhibitory effect on cofilin phosphorylation by LIMK2 might require the transient activation of LIMK2 by ROCK.

### SecPH inhibits LIMK2 phosphorylation of cofilin by preventing ROCK activation of LIMK2

**Figure 4:**
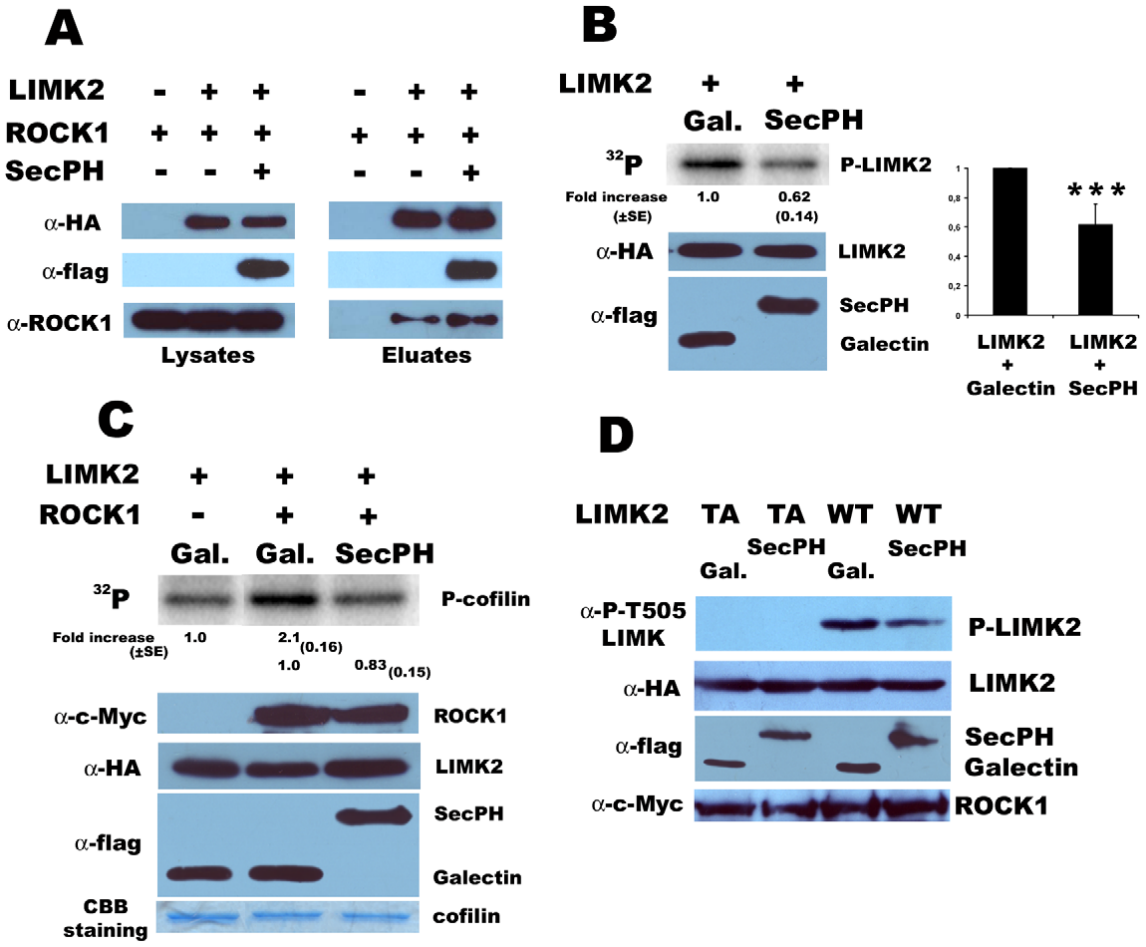
Mechanism of SecPH inhibition of LIMK2 kinase activity. *A. SecPH does not prevent LIMK2 from interacting with ROCK1*. Cells were cotransfected with HA-LIMK2, ROCK1 and SecPH or its empty parental plasmid. Lysates and anti-HA immunoprecipitates were subjected to immunoblotting. *B. SecPH affects LIMK2 phosphorylation.* Same as Figure 2B. Statistical significance was determined relative to control using one-way ANOVA (*** p<0.0001). *C. Inhibition of LIMK2 cofilin phosphorylation by SecPH in the presence of ROCK1.* Cells were cotransfected with ROCK1, LIMK2 and SecPH or Galectin-3 (a non-specific control protein). Immunoprecipitated HA-LIMK2 and GST-cofilin were used for the kinase assay. The kinase activity on cofilin of immunoprecipitated HA-LIMK2 from cells cotransfected with Galectin-3 was taken as 1.0. Each value represents the mean ± SE (standard error) of four independent experiments. The HA-immunoprecipitates were also submitted to immunoblotting and to coomassie blue staining. Lysates were also submitted to flag-immunoblotting. *D. SecPH affects LIMK2 T505 phosphorylation by ROCK1.* Cells were cotransfected with either LIMK2-TA or LIMK2-WT and SecPH or Galectin-3 (a non-specific control protein). Lysates were subjected to immunoblotting.

In an attempt to elucidate the mechanism of SecPH inhibition of cofilin phosphorylation by ROCK activated LIMK2, we postulated that SecPH might prevent LIMK2 from interacting with ROCK. To check this hypothesis, we cotransfected cells with HA-LIMK2, cMyc-ROCK1 and flag-SecPH or its empty parental plasmid. We then performed HA-immunoprecipitations. In these conditions, ROCK1 interacts with LIMK2 in the absence as well as in the presence of SecPH (Figure 4A). Therefore, SecPH does not disturb the interaction between ROCK1 and LIMK2.

Another attractive hypothesis is that SecPH might prevent the T505 phosphorylation of LIMK2 by ROCK.

This hypothesis was in accordance with ^32^P gels from the LIMK2 kinase assay on cofilin described in Figure 2B. Indeed, a slower mobility band compared to cofilin could be observed on ^32^P labeled gels. This band corresponded to LIMK2 molecular weight. So in this assay, we could also observe HA-immunoprecipitated LIMK2 phosphorylation. The intensity of LIMK2 phosphorylated band was decreased in the presence of SecPH (Figure 4B) indicating that SecPH might affect LIMK2 phosphorylation.

In order to know if this phosphorylation was mediated by ROCK activity on T505 of LIMK2, and was not due to LIMK2 autophosphorylation, we used anti-P-LIMK1 (T508)/LIMK2 (T505) antibodies. However, in conditions where cells were transfected with LIMK2, this antibody appeared to be non specific for P-LIMK2 (T505) detection. Indeed, a signal could be observed with the LIMK2-T505A mutant on lysate and even on LIMK2-T505A immunoprecipitate. To circumvent this problem, we cotransfected cells with LIMK2 and ROCK1, in order to enhance LIMK2-T505 phosphorylation. In these conditions, no signal was observed for LIMK2-T505A mutant (Figure 4D).

In these conditions *i.e.* when ROCK1 was cotransfected with LIMK2, SecPH was still able to inhibit cofilin phosphorylation by LIMK2, although HA-LIMK2 immunoprecipitates showed an increased kinase activity on cofilin (Figure 4C).

When cells were cotransfected with ROCK1, LIMK2 and SecPH, the signal detected with anti-phosphoT505 antibodies was significantly decreased compared to cells cotransfected with ROCK1, LIMK2 and a non-specific control protein, Galectin-3 (Figure 4D). These results suggest that SecPH affects LIMK2-T505 phosphorylation by ROCK1.

Altogether these data suggest that SecPH inhibition of cofilin phosphorylation by LIMK2 is due to SecPH inhibition of LIMK2 phosphorylation and therefore activation by ROCK1.

### SecPH affects ROCK kinase activity specifically with respect to LIMK2

Our results suggest that SecPH affects LIMK2 phosphorylation by ROCK. We next wondered if SecPH affects ROCK kinase activity in general or specifically with respect to LIMK2. In this purpose, we studied the kinase activity of ROCK on Myosin Light Chain (MLC), another ROCK substrate, in the presence or in the absence of SecPH.

**Figure 5:**
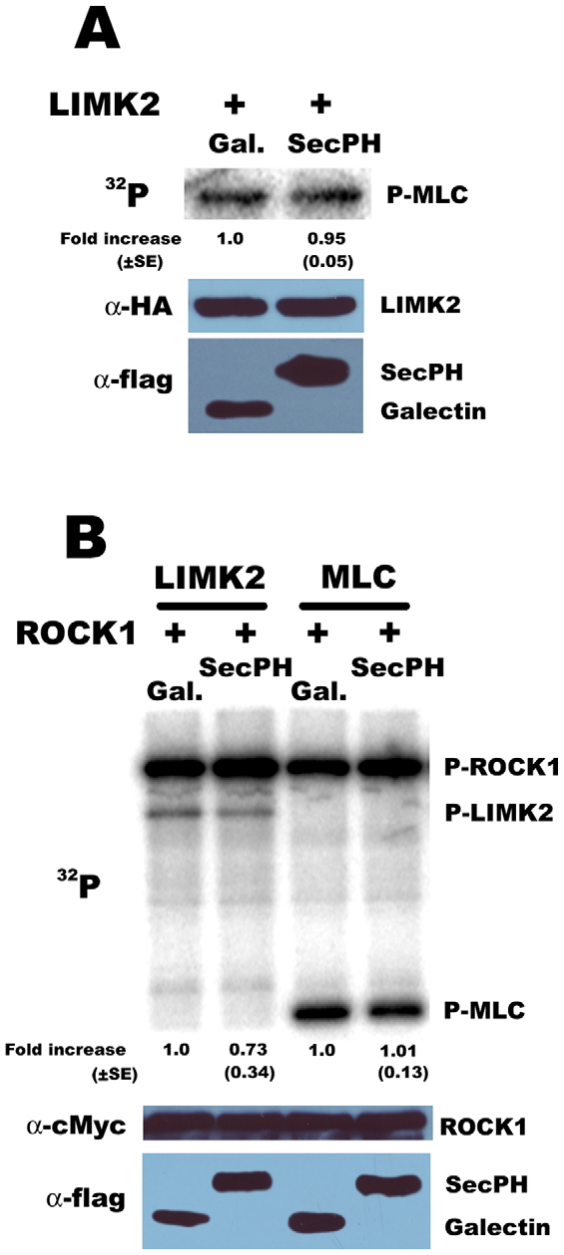
SecPH affects ROCK kinase activity specifically with respect to LIMK2. *A. MLC phosphorylation is not affected by SecPH.* Same as Figure 2B, except 0.5 µg of MLC were added in the kinase reaction mixture. Each value represents the mean ± SE (standard error) of two independent experiments. B. *MLC phosphorylation by ROCK-1 is not affected by SecPH.* Cells were cotransfected with ROCK1 and SecPH or Galectin-3. Immunoprecipitated c-Myc-ROCK1 was used for the kinase assay in the presence of recombinant LIMK2 or MLC (0.5 µg each). The kinase activity on LIMK2/MLC of immunoprecipitated c-Myc-ROCK from cells cotransfected with Galectin-3 was taken as 1.0. Each value represents the mean ± SE (standard error) of four independent experiments. Immunoprecipitates were also submitted to c-Myc-immunoblotting and lysates to flag-immunoblotting.

First, we repeated our kinase assay on HA-LIMK2 immunoprecipitates in the presence of SecPH or Galectin-3 as described in Figure 2B but we added MLC in the kinase reaction mixture. Indeed, as shown in Figure 4B, we know that in this assay, SecPH inhibits LIMK2 phosphorylation and we wanted to know if it was also the case for MLC. In these conditions, MLC phosphorylation did not seem to be affected by SecPH (Figure 5A).

Then, we transfected cells with c-Myc-ROCK1 and SecPH or Galectin-3. We measured the kinase activity of the anti-c-Myc immunoprecipitates but this time by using recombinant LIMK2 or MLC as substrates. As shown in Figure 5B, SecPH seemed to have no influence on MLC phosphorylation by ROCK1. In this experiment, SecPH inhibition of the recombinant LIMK2 phosphorylation was faint but this can be explained by the fact that the recombinant LIMK2 we used was already activated and phosphorylated. It has also to be noted that ROCK1 autophosphorylated during the assay and that SecPH did not seem either to inhibit this phosphorylation.

Altogether, these results suggest that SecPH inhibition of ROCK kinase activity is specific to LIMK2.

## Discussion

The human superfamily of small GTPases presents more than 150 members. Ras GTPases are the founding members of this family, which is divided into five main branches: Ras, Rho, Rab, Ran and Arf. Acting as molecular binary switches, these small GTPases regulate many major biological processes, such as cell cycle progression, cell survival, actin cytoskeleton organization, cell polarity and movement, and vesicular and nuclear transport. Extensive cross-talks between these different small GTPases have been demonstrated [1], [37], [38], [39].

In order to find new cross-talks between Ras GTPases and other small GTPases, we decided to focus on the RasGAP Nf1, which was already shown to integrate several signal transduction pathways. By a yeast two-hybrid screening, we identified LIMK2 as a new partner of the SecPH domain of Nf1. LIMK2 is a major kinase of the Rho/ROCK/LIMK2/cofilin pathway. Upon activation by ROCK, LIMK2 phosphorylates cofilin, resulting in its inactivation. Cofilin is a member of the ADF (actin depolymerizing factor) family. It promotes actin depolymerization at pointed ends and severes long actin filaments, which leads to a fast turnover of actin filaments [33], [34]. By inhibiting cofilin, LIMK2 plays a central role in the regulation of actin cytoskeleton reorganisation and thereby contributes to diverse cellular functions such as cell motility, morphogenesis, division, differentiation, apoptosis, neurite extension and oncogenesis.

We confirmed the interaction between LIMK2 and the SecPH domain of NF1 by coimmunoprecipitation experiments. Our results also suggest a molecular mechanism explaining the physiological relevance of this interaction. Indeed, by interacting with LIMK2, Nf1, *via* its SecPH domain, seems able to inhibit LIMK2 activation by ROCK and its subsequent activity on cofilin.

Our results are in accordance with data from a recent proteomic study on ROCK, whose goal was to identify new substrates of this kinase [40]. Nf1 and LIMK2 were found to be part of the ROCK complexome.

**Figure 6:**
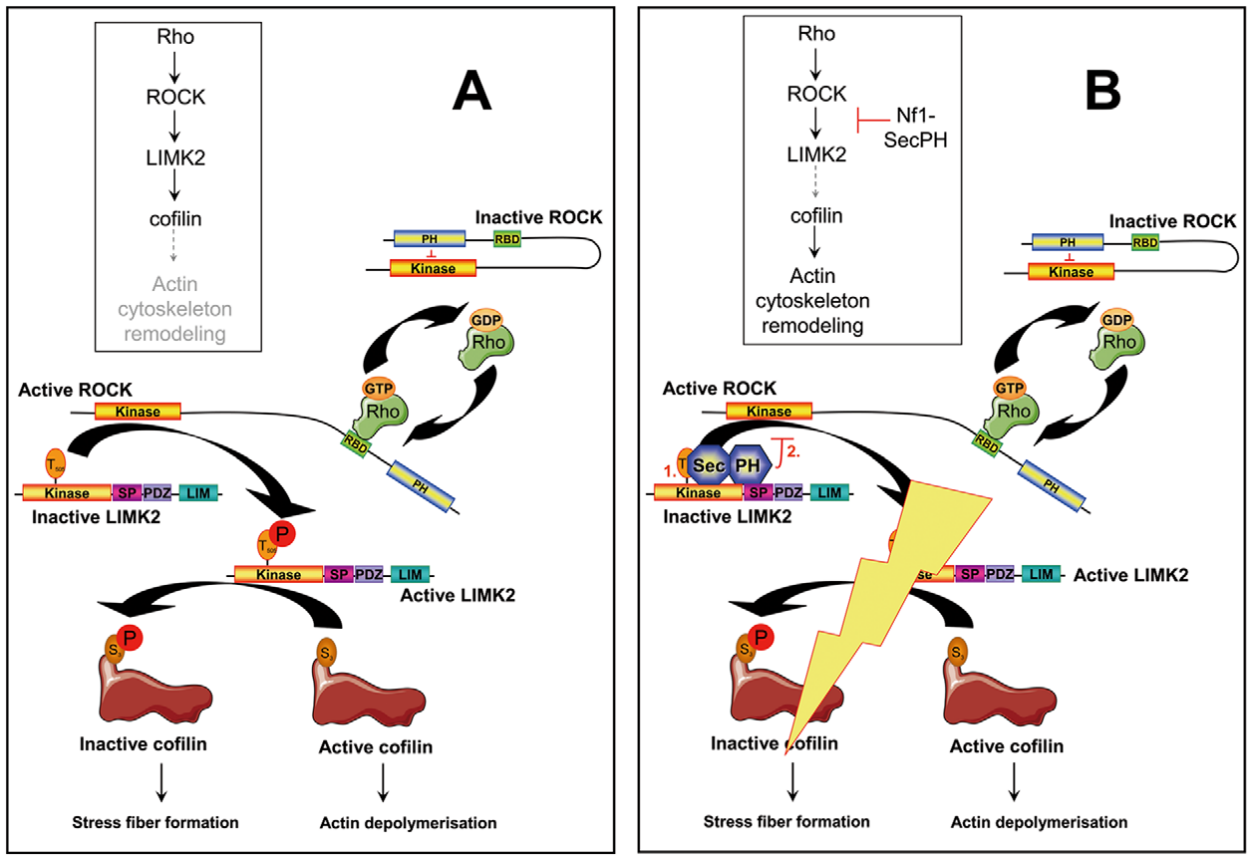
Schematic representation of our findings: A molecular connection between neurofibromin and the Rho/ROCK/LIMK2/cofilin pathway. *A.* Upon Rho activation *via* binding to its RBD (Rho Binding domain), ROCK activates LIMK2 by phosphorylation at its Thr505. Activated LIMK2 will then phosphorylate cofilin on its Ser3, resulting in its inhibition. An invasive phenotype is then observed with accumulation of actin stress fibers. *B. SecPH, a new inhibitor of this Rho/ROCK/LIMK2/cofilin pathway*. By interacting with LIMK2, SecPH prevents ROCK activation of LIMK2. Our data raises two possible hypotheses for this inhibition of ROCK activation of LIMK2 by SecPH: (1) by steric hindrance, SecPH hides Thr505 of LIMK2 from ROCK accessibility, (2) the PH domain of SecPH substitutes to the PH domain of ROCK by inhibiting the kinase activity of ROCK; this inhibition would specifically occur when ROCK and SecPH are simultaneously bound to LIMK2.

Our findings are also in good agreement with previous data from Ozawa *et al.* [29], who showed that Nf1 regulated actin cytoskeleton reorganization by inhibiting the Rho/ROCK/LIMK2/cofilin pathway. They performed *NF1* siRNA experiments on HeLa cells and observed an excessive actin stress fiber formation and an increase of P-cofilin due to LIMK2. However, they could not establish a direct molecular link between Nf1 and this pathway. Our data indicate that this missing link might be the interaction between LIMK2 and the SecPH domain of Nf1, although we cannot exclude another partner as we worked on immunoprecipitated and not recombinant proteins. We also went further into the understanding of the molecular mechanism of Nf1 inhibition of the Rho/ROCK/LIMK2/cofilin pathway by suggesting that Nf1-SecPH might prevent LIMK2 activation by ROCK and therefore prevent LIMK2 phosphorylation and inhibition of cofilin. SecPH inhibition of ROCK kinase activity seems to be specific to LIMK2, as phosphorylation of another ROCK substrate, Myosin Light Chain, is not affected by SecPH. It would be very interesting to go deeper into the understanding of the mechanistics of this process. Our study raised two hypotheses (Figure 6) : (1) we have shown that SecPH does not disrupt LIMK2/ROCK interaction (Figure 4A), however when SecPH binds to LIMK2, a steric hindrance might prevent ROCK from accessing its target residue, Thr505 of LIMK2 (2) when SecPH and ROCK bind to LIMK2, they get nearby, then the PH domain of SecPH might interact with the kinase domain of ROCK and inhibit it, thereby mimicking the action of the PH domain of ROCK in its inactive closed conformation. However, as we showed that SecPH has no intrinsic influence on ROCK kinase activity in general, this inhibition would specifically occur when ROCK and SecPH simultaneously interact with LIMK2. Further studies are currently in progress to bring an answer to these intriguing mechanistics.

Our results also corroborate previous data suggesting a role of Nf1 in actin cytoskeleton dynamics.

First of all, many of the clinical features of Neurofibromatosis type I, such as neurofibroma and glioma formation as well as learning disabilities, may be related to actin cytoskeletal organisation defect. Indeed, neurofibromas are composed of an aggregation of multiple cell types and they are infiltrated by surrounding hypermotile *Nf1*+/− mast cells (which secrete mediators that remodel the extracellular matrix and initiate angiogenesis). Both features are related to the deregulation of the actin cytoskeleton. Beyond the tumorigenic symptoms, NF1 patients frequently exhibit cognitive deficits. Along this line, regulation of actin cytoskeleton reorganization and the Rho/ROCK/LIMK/cofilin pathway have been shown to play a role in neuronal cell development [41], [42].

Secondly, enhanced migration and motility as well as abnormal actin cytoskeleton organization were observed in *Nf1* deficient or haploinsufficient Schwann cells, astrocytes and osteoclasts [43], [44], [45], [46]

Finally, in addition to the Rho/ROCK/LIMK2/cofilin pathway, several other specific signaling pathways were identified to connect Nf1 to actin cytoskeleton dynamics. *NF1* overexpression was shown to induce an increase in the expression levels of the Focal Adhesion Kinase (FAK) [24]. A direct interaction between Nf1 and FAK has also been described [27], suggesting a role of Nf1 in cell adhesion. Moreover, by interacting with syndecan-2, another adhesion protein, Nf1 mediates the activation of PKA, which phosphorylates two actin-associated proteins Ena and VASP, thus promoting actin polymerisation for the formation of filopodia and dendritic spines [28]. In addition to its negative regulation of the Rho/ROCK/LIMK2/cofilin pathway [29], Nf1 also independently negatively regulates the Rac1/Pak1/LIMK1/cofilin pathway [30]. In both cases, *NF1* depletion leads to an increased phosphorylation and consequently inhibition of cofilin, thus promoting actin stress fiber formation. Along the same line, the effect of the drug schweinfurthin A is most probably related to the role of Nf1 in the Rho/ROCK/LIMK2 pathway [47]. Recently, Nf1 was shown to activate Rho/ROCK/MLC pathway *via* cAMP/PKA signaling. These latter results seem in contradiction with the previous data described above and our findings, but can be easily reconciliated. Indeed, they were obtained specifically in neurons of the central nervous system and not in neurons of the peripheral nervous system, therefore they appear to be cell type specific [48].

From a signal transduction point of view, we identified a new cross-talk between Ras GTPases and another small GTPase, namely between Ras and RhoA.

About twenty years ago, several studies already suggested cross-talks between Ras and Rho signaling pathways [37], [49], [50]. The preliminary ideas limiting Ras to cell proliferation and Rho to actin cytoskeleton reorganization and considering these pathways as linear and disconnected became quickly obsolete. A complex scheme interconnecting the different small GTPase pathways was more accurate. Different approaches, sometimes combined, allowed to establish these connections: (i) the use of dominant negative and constitutively active versions of small GTPases (created through specific amino acid substitution); (ii) the use of transient activation or chemical drugs to stimulate or inhibit upstream regulators of small GTPases; (iii) more recently the use of RNAi and gene knockout models. Although these studies brought invaluable data on cross-talks between Ras and Rho, mainly *via* Rac, they suffered from a lack of molecular data. Indeed, most of the time they did not identify precisely the different proteins or the biochemical mechanisms involved in these connections. In parallel, studies based on GEFs and GAPs, the main regulators of small GTPases, also established connections between the different small GTPases. Sequence analysis showed that some GEFs, and in a lower extent a few GAPs, possess GEF or GAP domains for other small GTPases. By interacting with various specific partners, GAPs or GEFs appear to trigger different cell responses. As an example, when the GEF SOS is in complex with Grb2, it activates Ras, whereas when it is in complex with Eps8, it activates Rac [1], [51]. Special interest has focused on p120RasGAP, the first RasGAP identified. p120RasGAP was found to interact with several RhoGAPs, triggering different cell responses according to which RhoGAP is involved. p120RasGAP was found to interact with p190RhoGAP [49], [52]. It appears to recruit it to the cell periphery where it inhibits Rho [53]. p120RasGAP was also found to interact with p200RhoGAP. This interaction is required for p200RhoGAP to activate Ras, promoting cell growth and transformation [54]. Finally, p120RasGAP was found to interact with the DLC1 RhoGAP thereby inhibiting its GAP activity towards RhoA and resulting in RhoA activation [3].

In our present study, the RasGAP Nf1 does not interact with a RhoGAP but with a downstream effector of RhoA, LIMK2. By interacting with the SecPH domain of Nf1 Ras GAP, LIMK2 partially loses its kinase activity on cofilin. We have shown that this process is specific to LIMK2. Nf1 RasGAP-SecPH has no effect on LIMK1, the close related homolog of LIMK2. One may think that, in these conditions, cofilin may still be inactivated by LIMK1. However, another domain of the RasGAP Nf1, its pre-GAP region, has been shown to negatively regulate the Rac1/Pak1/LIMK1/cofilin pathway [30]. So, *via* two of its domains, the RasGAP Nf1 may coordinate the inhibition of both LIM kinases shutting down their activity on cofilin. The regulation of these two branches of the pathway by two independent domains suggests an independent regulation for these processes.

In conclusion, our study suggests a new connection into the complex network of small GTPase signaling. The RasGAP Nf1 might regulate the activity of a downstream effector of RhoA. Our results bring unprecedented details of the molecular mechanism that might be involved in this connection. Further characterization of the interactome of the different members of this network will certainly establish new cross-talks between small GTPases. A special effort on the molecular requirements of these interactions is needed for a better understanding of the role and the interconnections between each member in this network.

## Materials and Methods

### Materials

Antibodies against Nf1 (sc-67), ROCK1 (sc-6055), and LIMK2 (sc-8390) and anti-Nf1 beads were from Santa Cruz Biotechnology, Inc. Anti-flag M2 affinity gel, anti-flag M2 monoclonal antibody, flag-peptide and anti-HA and anti-c-Myc affinity gels were from Sigma-Aldrich Co. HA antibody was from Roche Applied Science and P-LIMK2 antibody from Cell Signalling Technology. Lipofectamine LTX was from Invitrogen, Opti-MEM from Gibco. Recombinant GST-fused cofilin was purchased from Upstate cell signalling Inc. Recombinant Myosin Light Chain and LIMK2 were from Calbiochem and Life Technologies, respectively. Plasmids used in this study are listed in Table 1.

**Table 1:** List of plasmids used in this study.

Plasmid	Description	Source/Reference
pBTM116	P*_ADH1_* -*LEXA TRP1* 2μ	[57]
pBTM116-SecPH	P*_ADH1_* –*LEXA-SecPH TRP1* 2μ	This study
pBTM116-TBD	P*_ADH1_* –*LEXA-Ira2 TBD TRP1* 2μ	This study
pACT2	2μ*LEU2*	Clontech
pACT2-TFS1	2μ*LEU2-TFS1*	This study
pACT2-LIMK1	2μ*LEU2-LIMK1*	This study
pACT2-LIMK2	2μ*LEU2-LIMK2-2a*	This study
p3XFlag-*myc*-CMV-24	P*_CMV_*-Flag	Sigma
p3XFlag-SecPH	P*_CMV_*-Flag-SecPH	This study
p3XFlag-Galectin-3	P*_CMV_*-Flag-Galectin-3	A. Legrand
pUC2SR-*myc*-LIMK2-2a	*LIMK2-2a*	K. Mizuno
pUC2SR-*myc*-LIMK2-2a-T505A	*LIMK2-2a*-T505A	K. Mizuno
pFC1-*myc*-hLIMK1	*LIMK1*	K. Mizuno
pcDNA3-(HA)_2_-LIMK2	P*_CMV_*-(HA)_2_-*LIMK2-2a*	This study
pcDNA3-(HA)_2_-LIMK1	P*_CMV_*-(HA)_2_-*LIMK1*	This study
pcDNA3-(HA)_2_-LIMK2-T505A	P*_CMV_*-(HA)_2_-*LIMK2-2a-T505A*	This study
pcDNA3-(HA)_2_-LIMK2-T505EE	P*_CMV_*-(HA)_2_-*LIMK2-2a-T505EE*	This study
pcDNA3-(HA)_2_-LIM-LIMK2	P*_CMV_*-(HA)_2_-*LIM*-*LIMK2-2a*	This study
pcDNA3-(HA)_2_-PDZ-LIMK2	P*_CMV_*-(HA)_2_-*PDZ*-*LIMK2-2a*	This study
pcDNA3-(HA)_2_-PDZ-SP-LIMK2	P*_CMV_*-(HA)_2_-*PDZ-SP*-*LIMK2-2a*	This study
pcDNA3-(HA)_2_-KIN-LIMK2	P*_CMV_*-(HA)_2_-*KIN*-*LIMK2-2a*	This study
pCAG ROCK1	*ROCK1-myc*	[58]
pET14-6his-SecPH	*6his-SecPH*	This study

### Two-hybrid screening

The two-hybrid system used was obtained from Clontech (Yeast Matchmaker). All media, buffers and methods used for yeast cells were adapted from previously described procedures [55], [56] and from the Clontech *Yeast Protocols Handbook*. pBTM116-SecPH encoding the SecPH domain of Nf1 fused in N-terminus to the LexA DNA-binding protein was transformed into the *Saccharomyces cerevisiae* strain L40 (MATa, *his3*Δ*200*, *trp1-901*, *leu2-3*, *112*, *ade2*, *LYS::(lexAop)_4_-HIS*, *URA3::(lexAop)_8_-lacZ*. The human foetal brain cDNA library, cloned in pACT2 (and fused in N-terminus to the activation domain of Gal4) was transformed in the Y187 strain (MATa, *ade2-101*, *met-his3-200*, *leu2-3*, *112*, *trp1-901*, *ura3-52*, *gal4Δ* *gal80Δ*, *URA3::GAL1*, *UAS-GAL1*, *TATA-lacZ MEL1*) and was purchased from Clontech. It contained 3.5 millions of independent clones. After mating of the two strains, 270 millions of interactions were tested and plated on restricted medium lacking leucin, tryptophan and histidin. Growing colonies were restreaked and tested for β-galactosidase activity, yielding 1464 positive clones that were collected and stored at −80°C in glycerol. 173 of these clones were identified by PCR-amplifying the corresponding prey fragments and sequencing them. Two of these clones encoded the full length LIMK2.

### Cell culture and transfection

HEK-293 (ATCC, CRL1573) and HeLa cells (ATCC, CCL-2) were cultured under 5% CO_2_ at 37°C in Dulbecco's modified Eagle's medium supplemented with 10% fetal calf serum. Cells were transiently transfected with 10 ug of plasmid/100-mm dish with Lipofectamine LTX according to manufacter's recommendations. Experiments were performed 24 h to 48 h after transfection.

### Immunoprecipitation

HEK-293 cells were transiently transfected with expression plasmids as described above, and cultured for 24 to 48 h. Cells were lysed in 0.5 ml of lysis buffer (50 mM Tris/HCl, pH 7.5, 100 mM NaCl, 5 mM EDTA, 0.1% Triton X-100, 50 mM NaF, 10 mM sodium pyrophosphate, 1 mM Na_3_VO_4_, 20 mM p-nitrophenyl phosphate, 20 mM β-glycerophosphate, 10 µg/ml aprotinin, 0.05 µg/ml okaidic acid, 1 µg/ml leupeptin, and 1 mM PMSF), and incubated on ice for 10 min. After centrifugation, the supernatants were incubated for 3 h at 4°C either with anti-HA affinity gel for HA-LIMK2 or its derivatives or with anti-flag M2 affinity gel for SecPH and Galectin-3 or with anti-Nf1 affinity gel for endogenous Nf1. Beads were washed five times with lysis buffer. HA-LIMK2 or its derivatives and Nf1 were eluted by Laemmli sample buffer and flag-SecPH or flag-Galectin-3 was eluted by incubating the beads for 30 minutes on ice with 0.2 mg/ml of the flag peptide.

### 6His-SecPH purification

6His-SecPH was expressed in *Escherichia coli* using the pET14 plasmid. The protein was purified from the bacterial extract by using TALON Metal Affinity Resin (Clontech). Elution was performed with 100 mM imidazole. Protein concentration was measured by using Bradford method.

To test the interaction between 6His-SecPH and LIMK2, HA-LIMK2 was immunoprecipitated as described in the previous section. Immunoprecipitated beads were then incubated with 6His-SecPH (12 µg) in lysis buffer for 2 hours, and further washed three times with lysis buffer, and then eluted by Laemmli sample buffer. For the negative control, the anti-HA immunoprecipitation was performed on lysates of HEK-293 cells transfected with the pcDNA3 empty plasmid (parental plasmid of HA-LIMK2).

### Kinase assay

Immunoprecipitates bound to HA- or flag-beads, as described above, were washed twice with lysis buffer and then three times with kinase buffer (50 mM HEPES-NaOH pH 7.5, 150 mM NaCl, 5 mM MgCl_2_, 5 mM MnCl_2_, 50 mM, NaF, 1 mM Na_3_VO_4_, 20 mM β-glycerophosphate, 1 µg/ml leupeptin, and 1 mM PMSF). HA-LIMK2 or its derivatives were used bound on beads. Flag-SecPH or Flag-Galectin-3 were eluted by incubating the beads with the flag peptide. Immunoprecipitates were incubated for 20 min at 30°C in 22 µl of kinase buffer containing 50 µM ATP, 5 µCi of γ[^32^P]ATP (3,000 Ci/mmol) and 2.5 µg of GST-fused cofilin. The reaction was terminated by heating 5 minutes at 90°C in Laemmli sample buffer. Samples were then subjected to SDS-PAGE, and analyzed by autoradiography.

### Cell staining

HeLa cells were fixed with 4% paraformaldelyde in PBS for 20 min and permeabilized with 0.5% Triton-X100 in PBS for 15 min at room temperature. After blocking with 1% fetal calf serum in PBS for 30 min, these cells were incubated with anti-HA, anti-flag antibodies for 1 h and subsequently with FITC-conjugated anti-rat IgG and AlexaFluor647-conjugated anti-mouse IgG, respectively, and simultaneously with AlexaFluor568-conjugated phalloidin for 1 h. The cells were then washed three times with PBS, mounted on glass slides, and then analyzed by confocal microscopy using a Zeiss Axiovert 200 M microscope coupled with a Zeiss LSM 510 scanning device (Carl Zeiss Co. Ltd., Iena, Germany).
